# Practical Implementation of the BLW Method During the Expansion of the Infant Diet—A Study Among Polish Children

**DOI:** 10.3389/fnut.2022.890843

**Published:** 2022-05-24

**Authors:** Agnieszka Białek-Dratwa, Elżbieta Szczepańska, Paulina Trzop, Martina Grot, Mateusz Grajek, Oskar Kowalski

**Affiliations:** ^1^Department of Human Nutrition, Department of Dietetics, Faculty of Health Sciences in Bytom, Medical University of Silesia in Katowice, Bytom, Poland; ^2^Department of Dietetics, Faculty of Health Sciences in Bytom, Medical University of Silesia in Katowice, Bytom, Poland; ^3^Faculty of Health Sciences in Bytom, Medical University of Silesia in Katowice, Bytom, Poland; ^4^Department of Public Health, Faculty of Health Sciences in Bytom, Medical University of Silesia in Katowice, Bytom, Poland; ^5^Department of Public Health Policy, Faculty of Health Sciences in Bytom, Medical University of Silesia in Katowice, Bytom, Poland

**Keywords:** Child Nutrition, Baby Led Weaning (BLW), expanding the diet of infants, complementary feeding, child diet

## Abstract

The aim of the study was to verify the knowledge of mothers of children under 3 years of age about the Baby Led Weaning (BLW) feeding model and their practical implementation of this method. The study involved 761 mothers and their children. After analysis of the inclusion and exclusion criterion, the information provided by women 699 aged 21–48 years was included in the final data analysis. In the study group, most children were breastfed for 6 months to 1 year (*n* = 256, 36.7%), 1 year to 2 years (*n* = 179, 25.6%) and over 2 years (*n* = 71, 10.2%). Starting dietary expansion before 17 weeks of age was implemented in 47 (6.7%) children, between and 17–26 weeks of age in 328 (46.9%) children, and after 26 weeks of age in 324 (46.3%) children. Feeding food and dishes from the family table was practiced by 518 (74.1%) mothers. Spoon-feeding was practiced by 529 (75.6%) children, 157 (22.4%) children were fed this way sometimes. Taking into account the above data, feeding with the BLW method was used in 170 children (24.2%). In the examined group of mothers the use of the BLW method in feeding their children, especially during diet expansion, was declared by 408 women (74.8%). The child's independent decision concerning what the child will eat and what is according to the BLW method is accepted by 434 (62.1%) mothers. Among the positive aspects of using the BLW method, the women surveyed indicated the child's independence, while among the disadvantages, the omnipresent mess and chaos when eating meals.

## Introduction

In the postnatal period, and especially in the first 33 months of a child's life, nutrition is a form of synbiotic directed at the gastrointestinal tract, more specifically at the intestinal microbiota, with a regulatory effect on the gut-brain axis. Because of this issue, it is important to shape pro-healthy eating habits in children (1). The recommended way of diet expansion, which at the same time introduces a lot of controversies, is the *Baby Led Weaning* (BLW) feeding model (2, 3). The Polish Society of Gastroenterology, Hepatology and Child Nutrition (PTGHiZD) defines this method of feeding as feeding with the omission of mushy consistency served with cutlery, with the exclusive implementation of solid consistency eaten by an infant on its own (4). It is a method of expanding and supplementing feeding between 17 and 26 weeks of age (4). According to the World Health Organization (WHO), a baby should be breastfed exclusively with breast milk for the first 6 months. After the introduction of complementary foods, WHO and ESPGHAN recommend continuing breastfeeding for as long as desired by mother and child (5, 6). WHO recommends starting the introduction of complementary foods around the sixth month of a child's life (6). On the other hand, ESPGHAN and PTGHiZD recommend starting the introduction of solid foods after the 17th and no later than the 26th week of a child's life. These two different positions may confuse parents as to when they should start expanding the child's diet (4, 5). The introduction of new products to the infant's diet also aims to prepare the infant for a more varied diet later in life, including learning tastes and textures (7, 8). In most infants, between 17 and 26 weeks of age, the ability to accept solid foods matures. It is not until the infant is 17 weeks old that the gastrointestinal tract and kidneys are mature enough for the infant to assimilate non-dairy foods. The swallowing reflex and the ability to accept puréed food from a spoon appear between 4 and 6 months of age. The introduction of foods before the age of 3–4 months also carries a risk of allergy (5). Infants between 17 and 26 weeks of age develop the ability to sit with support, acquire neuromuscular maturity that allows them to control head and neck movements, and to eat from a spoon. At this time, the reflex to remove foreign bodies from the mouth, which made feeding with food other than liquid difficult, ceases (4–6). All these aspects allow for the introduction of feeding with the BLW method. Numerous parents' doubts about its use, not based on scientific reports (Evidence-Based Medicine—EBM), focus on the fear of nutritional deficiencies (especially of microelements, e.g., iron, zinc, vitamin B_12_), growth retardation, disorders of psychomotor development, and choking defined as a life-threatening condition involving partial or complete obstruction of the upper airways. Often their concerns also relate to wasting food, as well as the appearance of the environment after the child has eaten a meal on their own (2, 4). In the BLW method, eating together as a family and at meal times is important. This approach can give the infant more control over food intake. This can lead to better eating patterns and reduce the risk of overweight and obesity. Given the self-selection of parents and infants who currently use the BLW method, and the limited observational data available however, it is not possible to draw conclusions. Furthermore, there is a lack of data on whether infants who are traditionally spoon-fed receive sufficient nutrients, including energy and iron, or whether they consume more or they consume a greater variety of foods. ESPHGAN indicates that these issues should be investigated in RCTs. A modified version of BLW called BLISS—emphasizes the importance of introducing iron- and energy-rich energy-rich complementary foods and the avoidance of foods that may present choking hazards. A small observational pilot study suggests that this approach had some benefit in increasing iron-rich foods consumed by infants (4, 5). In a study by Neves et al. (9) note that there is still insufficient scientific and quality evidence to confirm that the BLW method is the most appropriate form of feeding into the diet of infants. Health professionals from New Zealand and Canada were concerned about the potential for energy and iron deficiencies in BLW children, which can cause disruption to children's growth and normal development (9–11).

In response to these concerns, suggestions for safe products adapted to the age of the child can be made, based on the experience of a specialist in clinical and pediatric dietetics (12) (Table 1).

**Table 1:** Proposal of implementation of complementary products.

**6–7 months of the child's life**	**8–12 months of the child's life**
• Soft vegetable pieces in cooked form (potato, carrot, courgette, pumpkin, beetroot, cauliflower, broccoli)	• Meals of a lumpy consistency
• Puree of the above vegetables • Pureed fruit (peach, apricot, pear, banana, apple)	• Food served in pieces in the child's hand
• Groats from gluten cereals and gluten-free cereals in thick consistency • Protein in the form of meat—poultry, fish • Vegetable oils, groundnuts, grains, seeds • Egg yolk • Fermented milk products (buttermilk, kefir, natural yogurt) • Mineral water, still in unlimited quantity	• The process of continuing the model of expanding nutrition in the form of new foods from the group of products such as fruit, vegetables, groats, pasta, bread, egg white, nuts, etc.

*Own elaboration based on pediatric Dietetics, chapter 2.5 Expanding the infant's diet in practical aspects. Medical University of Silesia Publishers, 2020 (12)*.

Another method aimed at partially alleviating parents' anxiety is *Baby-Led Introduction to Solids* (BLISS). This modification consists in introducing products adapted to the child's developed body motor mechanism, followed by biting, chewing and swallowing, and fortification in the presence of iron. When implementing both dietary models, it is important to keep in mind the sensory sensitivity of the child without leading to the appearance of aversion (13, 14). The sensory diversity of children is an important issue, so three criteria referring to the level of inclination toward the child's food preferences should be taken into account (15) (Table 2). BLISS resources are consistent with the BLW philosophy but address three key concerns that some health professionals have expressed about BLW: inadequate iron intake, choking and stunted growth. By: checking foods before serving to make sure they are soft enough, avoiding foods that form a crumb in the mouth, foods should be at least as long as the baby's fist, at least one side of the food, the baby's sitting position so they sit up straight while eating—never leaning back, that an adult is always with the baby, and that the baby does not put whole foods in their mouth—the baby needs to do this at their own pace and under their own control (16).

**Table 2:** Examples of sensory diversity among children.

The tendency to the child's food preferences	(1) Food habituation to milk consumption with a concomitant desire to consume introduced new products (vegetables, fruit, eggs, porridge, meat) occurs in breastfed and artificially mixed children
	(2) Eating habits focusing exclusively on milk consumption through breastfeeding and the introduction of artificial mixtures—modified milk, with aversion to new complementary products
	(3) Food habits focusing on food aversion toward milk in the form of a bottle and breastfed, but sensory inclination toward complementary products initially in thick form served with a spoon (porridge, soups, fruit), then in solid form (pieces of products).

*Own elaboration based on what do happy toddlers eat? Nutritional Publishers, 2020 (15)*.

The key influence on the positive effects of their application and the introduction of complementary foods is the clinical condition of the infant. Simultaneously occurring symptoms of food neophobia or food selectivity and the period of product administration (preferably after 6 months of age), the acceptance of known foods (facilitates the introduction of new products), the method of breastfeeding (brings many benefits), as well as the prenatal period in the past influence the way of expanding the diet of a small child. According to the concept of metabolic programming, already the diet of a pregnant woman shows an influence on the composition of amniotic fluid, and this, in time, will influence the way of diet expansion in an infant and his/her willingness to learn new tastes (17–20). The use of dietary expansion methods in the form of BLW or BLISS predisposes to the appearance of elements of dietoprophylaxis and health prevention in the later stages of a child's adolescence. They include low risk of metabolic diseases (overweight, obesity, hybrid diabetes), correct regulation of the hunger and satiety center—control of physiological hunger, developed on a differentiated, rational level eating habits by regulating the food given by the infant independently. Therefore, it is the toddler who will decide on the number of portions in conditions devoid of emotional pressure by the parents, with all the hygienic and safety rules monitored by them, maintained during the meal. It should be mentioned that among the pro-health elements there is also the education of parents and the use of the family table method. Next, it seems important to shape motor skills among infants and an independent relationship with food, as well as to stimulate the child's masticatory system (21–23).

The aim of the study was to verify the knowledge of the Baby Led Weaning feeding model among mothers of children up to 3 years of age and their practical implementation of this method.

## Materials and Methods

### Test Group

An exploratory cross-sectional study was conducted from April to June 2021 among randomly selected 761 mothers and their children, aged up to 3 years, residing in Upper Silesia. Mothers were recruited at randomly selected nurseries and pediatric clinics during mandatory vaccination visits. A list of public and private nurseries has been compiled, as well as a list of pediatric clinics where vaccinations are carried out for children aged 0–3 years—these establishments are both public and non-public and have a contract with the Narodowy Fundusz Zdrowia (National Health Fund), which finances vaccinations in Poland. We have defined randomization nurseries and pediatric outpatient clinics. 10 nurseries and 10 pediatric outpatient clinics were drawn from the list. In each nursery and pediatric clinic 40 parents of children aged 0–3 years were invited to participate in the study. All participants were informed about the purpose of the study, voluntary participation in the study, and anonymity, and were asked to accept the data sharing policy. After considering the inclusion and exclusion criteria, information collected from 699 women was included in the final analysis.

Due to the nature of the study, consent was sought from the Bioethics Committee of the Medical University of Silesia in Katowice. By the Resolution No. KNW/0022/KB1/151/I/11 the Bioethics Committee issued a positive opinion on the project. The study is in accordance with the Declaration of Helsinki.

### Rationale for the Selection of the Group

According to the current Polish law, after giving birth a mother is entitled to a maternity leave of 20 weeks for one child, 31 weeks for twins, 33 weeks for three children, 35 weeks for four children, 37 weeks for five and more children (24). After this time, parental leave can be taken, which lasts 32 weeks in the case of the birth of one child and entitles both parents to take it. Reports from the Social Insurance Institution indicate that in Poland, from January to May 2021, more than 246,000 parents, including only 1,900 men, benefited from maternity benefits for the period of parental leave (25). Therefore, mothers were invited to the study on infant diet expansion, as they are the ones who mostly spend time with their children and are responsible for expanding their diet.

### Inclusion and Exclusion Criteria

Inclusion criteria were: female sex, having a child aged 0–36 months (up to 3 years) of age, consenting to the study, and completing the questionnaire correctly and completely.

On the other hand, the criteria for exclusion from the study were: lack of consent to participate in the study, incorrectly completed questionnaire, including non-response to questions, and the child's age above 36 months.

### Research Tool

The research tool was a survey questionnaire, which consisted of several parts. The survey was conducted personally by qualified interviewers who were thoroughly trained on how to fill in the questionnaires—they were students of Dietetics at the Medical University of Silesia in Katowice. The questionnaires were filled in and entered by the interviewers in electronic form using tablets. The first part was a metric that asked women about their age, place of residence, education, height, and body weight. In the study conducted at the pediatric outpatient clinic, all children had their weight and length/height measured by nurses. In the nursery, on the other hand, weight and length/height measurements were taken by staff employed in the institution. Mothers, on the other hand, entered their own weight and height. There were also questions about the current work situation, education, marital status, number of children, and age of the youngest child.

The next part of the questionnaire concerned the basic knowledge about expanding the diet of a child and expanding the diet of a child using the BLW method. The part of the questionnaire concerning the method of extending the diet of a child included questions about the time of starting and the method of extending the diet of a child, the use of the BLW method, and indicating the advantages and disadvantages of this method or risks in independent eating by a child. At the same time, the questionnaire included questions about giving food to the child from the family table, feeding the child with a spoon, allowing the child to decide independently what and how much to eat, the order of introducing particular food products to the diet, the consistency of meals from which the expansion of the child's diet should begin, the type of thermal processing used to prepare meals, the time when the child received sweets for the first time.

The questionnaire was based on current dietary recommendations for the group of the youngest children and the method of dietary expansion developed by PTGHiZD (4). A pilot study was conducted on a group of 50 mothers in order to validate the questionnaire and to check the relevance and acceptability of the questions included in it. Reproducibility of responses was tested by comparing responses in the same group of subjects. Pilot study 2 took place 1 month after the pilot study to avoid freshness effects. To assess the reproducibility of the results obtained with the questionnaire used, the *x* parameter (Kappa) was calculated for each question in the questionnaire (results obtained in the pilot study and pilot study 2). For 67.3% of the questions, a very good (*x* ≥ 0.80) concordance of responses was obtained, and for 28.4% of the questions a good (0.79 ≥ *x* ≥ 0.60) concordance of methods was obtained. For only 4.3% of the questions in the questionnaire analyzed, the concordance between the results obtained at baseline and in the repeat survey was moderate (*x* < 0.59).

Also analyzed, Cronbach's α coefficient for the normalization sample was 0.87, which indicates high reliability and repeatability of the questionnaire. The pilot study allowed us to validate the questions included in the questionnaire. Cronbach's alpha for the relevant part of the study was estimated at 0.86.

### Statistical Analysis

Statistical analysis was performed using the software in Statistica v. 13.1 (StatSoft Inc., Tulsa, OK, USA). Non-parametric tests were used for statistical elaboration. Differences between groups were tested by Pearson's Chi-square test, with Fisher's exact, Yates, and the level of statistical significance was taken at *p* < 0.05.

## Results

### Characteristics of the Study Group of Mothers and Their Children

The characteristics of the study group of mothers are shown in Table 3. The largest group were mothers aged 31–35 years (*n* = 261; 37.4%), with a mean age of 33.9 ± 4.7 years. In the group of surveyed mothers, women living in a city with more than 500 000 inhabitants were *n* = 644; 92.1%. Higher education among the mothers studied was *n* = 613; 87.8%. On the other hand, marital status—married had *n* = 559; 80.1%. Analyzing body weight composition, normal body weight was *n* = 459; 65.7%. Most mothers in the study group had two children (*n* = 331; 47.4%).

**Table 3:** Characteristics of the study group of mothers.

	***n***	**%**
**Age of mothers**		
21–25 years	18	2.6
26–30 years	153	21.8
31–35 years	261	37.4
36–40 years	227	32.5
Over 40 years	40	5.7
**Place of residence of mothers**		
City over 500 thousand inhabitants	644	92.1
City with 100–500 thousand inhabitants	26	3.7
City with 20–100 thousand inhabitants	14	2.0
Village of	15	2.2
**The educational level of mothers**		
Higher	613	87.8
Averages	71	10.2
Professional	9	1.3
Basic	5	0,7
**Marital status of mothers**		
Married	559	80.1
Miss	97	13.9
Informal relationship	25	3.6
Divorced	16	2.3
Widow	1	0.1
**Maternal body weight by BMI**		
Underweight	29	4.2
Correct body weight	459	65.7
Overweight	136	19.5
Obesity	52	7.4
No data available	22	3.2
**Number of children**		
One	250	35.8
Two	331	47.4
Three or more	117	16.8

The characteristics of the studied group of children and the diet of this group are presented in Table 4. The largest group were children aged 2–2.5 years (*n* = 299, 42.7%), followed by 1–1.5 years (*n* = 168, 24.0%) and 2.5–3 years (*n* = 118, 16.9%). Most children were fed breast milk for 6 months to 1 year (*n* = 256, 36.7%), followed by 1–2 years (*n* = 179, 25.6%). Dietary expansion before 17 weeks of age was implemented in 47 (6.7%) children, between 17 and 26 weeks of age in 328 (46.9%) children (ESPGHAN and PTGHiZD recomendation) and after 26 weeks of age in 324 (46.3%) children. Feeding food and dishes from the family table was practiced by 518 (74.1%) mothers. Spoon-feeding was practiced by 529 (75.6%) children and 157 (22.4%) were occasionally fed in this way. Considering the above data, BLW feeding was used in 170 children (24.2%).

**Table 4:** Characteristics of the studied group of children and their diet.

	***n***	**%**
**Age of child**		
0–0.5 years of age	38	5.4
0.5–1 year old	32	4.5
1–1.5 years old	168	24.0
1.5–2 years	44	6.3
2–2.5 years	299	42.7
2.5–3 years	118	16.9
**Length of breastfeeding by mothers**		
Less than a month	42	6
1–2 months	46	6.6
3–4 months	56	8
5–6 months	47	6.7
6 months to 1 year	256	36.7
1 to 2 years	179	25.6
Over 2 years	71	10.2
**Time to start expanding the diet**		
Before week 17	47	6.7
Between 17 and 26 weeks	328	46.9
After 26 weeks	324	46.3
**Serving of products/dishes from the family table**		
Yes	518	74.1
Sometimes	153	21.9
Not	28	4.0
**Feeding the baby with a spoon**		
Yes	529	75.6
Sometimes	157	22.4
Not	13	1.8

### Knowledge and Methods to Expand the Child's Diet

In the part of the study concerning the knowledge of dietary expansion with the BLW method 545 (78.0%) women recognized this method of dietary expansion and demonstrated its knowledge. The main source of knowledge on this subject for the participating women was the Internet (*n* = 244; 43, 96%), followed by friends (*n* = 117; 21.8%), books (*n* = 75; 13.51%), internet forums for mothers and professional literature, respectively (*n* = 35; 6.31%) and (*n* = 34; 6.13%). Respectively, 14 (2.52%) and 13 (2.34%) mothers received information on the BLW method from their doctor or in the birthing school (Table 5). Of the 545 mothers who declared knowledge of the BLW method, 480 (88.1%) considered this method as appropriate.

**Table 5:** Mothers' nutritional knowledge of BLW.

	***n***	**%**
**Knowledge of the BLW method as a means of dietary expansion**		
Yes	545	78.0
Not	154	22.0
**Source of information about the BLW method**		
Internet	244	43.96
From friends	117	21.08
From books	75	13.51
From a forum for mothers	35	6.31
From professional literature (e.g., Pubmed)	34	6.13
Other sources of knowledge	15	2.70
From the doctor	14	2.52
From the birthing school	13	2.34
From the nutritionist	4	0.72
From the midwife	3	0.54

The use of the BLW method in feeding their children, especially during diet expansion, was declared by 408 (74.8%) women. The implementation of this model of feeding by the examined women was induced by the child's independence, due to the accelerated psychomotor development (*n* = 202; 37.0%), the wish to familiarize the child with various tastes and to shape the sensory diversity (*n* = 55; 10.1%), the desire to broaden family ties by using the method of eating from the family table (*n* = 50; 9.1%). The observations made by mothers during the process of dietary expansion with the BLW method were mainly the occurrence of mess (*n* = 147, 36.0%), the necessity to feed the child (*n* = 15, 3.7%), food wastage (*n* = 13, 3.2%), lack of maturity of the child for the BLW method (*n* = 7, 1.7%) and treating food as a game (*n* = 3, 0.7%). As many as 45 (11.1%) mothers indicated the occurrence of a food choking incident.

Independent eating by children was indicated by 563 (80.5%) mothers. The child's independent decision concerning what to eat, which is in accordance with the BLW method, is accepted by 434 (62.1%) mothers. 39 (5.6%) mothers do not allow their children to choose what to eat and 15 (2.15%) of them do not allow their children to decide how much to eat (Table 6).

**Table 6:** Use of the BLW method and children's independence in making feeding decisions.

	***n***	**%**
**Using the BLW method in feeding their children (*****n*** **=** **545; 100%)**		
Yes	408	74.8
Not	137	25.1
**Observed behavior during the application of the BLW method**		
**(*****n*** **=** **408; 100%)**		
No disadvantages of using the BLW method	178	43.6
The mess	147	36.0
Choking	45	11.0
The need for feeding	15	3.7
Waste of food	13	3.2
Lack of maturity on the part of the child to use the BLW method	7	1.7
Treating food as play	3	0.7
**Self-catering by the child**		
Yes	563	80.5
Sometimes	116	16.6
Not	20	2.9
**The child's own decision on what to eat**		
Yes	434	62.1
Sometimes	226	32.3
Not	39	5.6
**Child's own decision on how much to eat**		
Yes	597	85.41
Sometimes	87	12.45
Not	15	2.15

As declared by mothers, the basic products which were introduced to the diet with the BLW method included: carrots (*n* = 646; 92.4%), potatoes (*n* = 531; 75.9%), apples (*n* = 503; 71.9%) and bananas (*n* = 420; 60.0%). Less frequently, cereals, turkey, eggs, yogurt, bread, chicken, fish, and tomato were introduced first (Table 7).

**Table 7:** Products implemented by the BLW method (multiple choice question).

		***n***	**%**
Most commonly implemented nutritional products	Carrot	646	92.4
	Potatoes	531	75.9
	Apple	503	71.9
	Banana	420	60.0
Less frequently implemented nutritional products	Groats	197	28.1
	Turkey	137	19.6
	Eggs	136	19.4
	Yogurts	114	16.3
	Bread	105	15.0
	Chicken	102	14.5
	Fish	98	14.0
	Tomato	59	8.4
	Cucumber	46	6.5

The predominant forms of thermal processing that the products introduced into the child's diet were given were: cooking (*n* = 699; 100%), steaming (*n* = 455; 65.1%), baking (*n* = 465; 66.5%), stewing (*n* = 421; 60.2%).

### Socio-Economic Factors and the Use of the BLW Method

The relationships between the analyzed socioeconomic factors and the use of BLW as a method of child feeding are presented in Table 8. Neither education (*p* = 0.22), age of the examined child (*p* = 0.43), nor mother's body weight (*p* = 0.57) affected the decision about the use of BLW. On the other hand, a significant correlation was found between the duration of breastfeeding and the use of BLW (*p* = 0.003); the longer the mother breastfed, the more often she declared the use of BLW when expanding the child's diet.

**Table 8:** Relationship between BLW use and sociodemographic factors.

**Feature**	**Variable**	**Uses BLW**	**Does not use BLW**	**Materiality level**
		***n* (%)**	***n* (%)**	
Education	Higher	380 (93.1)	122 (89.1)	*P* = 0,22
	Medium	23 (5.6)	14 (10.2)	
	Vocation	4 (1.0)	0 (0.0)	
	Basic	1 (0.2)	1 (0.7)	
Length of breastfeeding	Less than 1 month	21 (5.1)	9 (6.6)	*P* = 0,003
	1–2 months	12 (2.9)	15 (10.9)	
	3–4 months	21 (5.1)	10 (7.3)	
	5–6 months	23 (5.6)	14 (10.2)	
	6–12 months	154 (37.7)	50 (36.5)	
	12–24 months	129 (31.6)	26 (19.0)	
	Over 24 months	48 (11.8)	13 (9.5)	
Age of child tested	0–0.5 years	22 (5.4)	6 (4.4)	*P* = 0,43
	0.5–1 year	18 (4.4)	11 (8.0)	
	1–1.5 years	110 (27.0)	31 (22.6)	
	1.5–2 years	25 (6.1)	7 (5.1)	
	2–2.5 years	169 (41.4)	61 (44.5)	
	2.5–3 years	64 (15.7)	21 (15.3)	
Mother's BMI	Underweight	21 (5.1)	5 (3.6)	*P* = 0,57
	Normweight	279 (68.4)	87 (63.5)	
	Overweight	77 (18.9)	29 (21.2)	
	Obesity	23 (5.6)	13 (9.5)	
	Data not available	8 (2.0)	3 (2.2)	
	Total	408 (100)	137 (100)	

### Declarations on the Use of the BLW Method vs. Its Practical Application

At the same time, it was examined to what extent mothers' declarations concerning the application of the BLW method were reflected in the actual application of the principles of this method: such as giving the child products/meals from the family table to eat (*p* = 0.00), feeding the child with a spoon (*p* = 0.00), allowing the child to eat a meal on its own (*p* = 0.00), allowing the child to decide on its own how much to eat (*p* = 0.00), and what to eat (*p* = 0.00) (Table 9).

**Table 9:** Assumptions of the BLW method used and not used in the group of mothers declaring and not declaring to use the BLW method.

**Feature**	**Variable**	**Uses BLW**	**Does not use BLW**	**Materiality level**
		***n* (%)**	***n* (%)**	
Giving the child food from the family table to eat	Yes	312 (76.5)	83 (60.6)	*P* = 0,00
	Sometimes	89 (21.8)	39 (28.5)	
	Not	7 (2.9)	15 (10.9)	
Feeding the baby with a spoon	Yes	251 (61.5)	31 (22.6)	*P* = 0,00
	Sometimes	145 (35.5)	6 (4.4)	
	Not	12 (2.9)	0 (0.0)	
Allowing the child to eat alone	Yes	358 (87.7)	87 (63.5)	*P* = 0,00
	Sometimes	52 (12.7)	43 (31.4)	
	Not	2 (0.5)	7 (5.1)	
Allowing the child to decide for him/herself what to eat	Yes	310 (76.0)	48 (35.0)	*P* = 0,00
	Sometimes	95 (23.3)	67 (48.9)	
	Not	3 (0.7)	22 (16.1)	
Allowing the child to decide how much to eat	Yes	366 (89.7)	112 (81.8)	*P* = 0,00
	Sometimes	39 (9.6)	19 (13.9)	
	Not	3 (0.7)	6 (4.4)	
	Total	408 (100)	137 (100)	

Despite the declaration of using the BLW method in the process of expanding the child's diet, the majority of mothers and their children used the mixed method. In the examined group of mothers, some of them declared that they did not use the BLW method, but they still implemented some of its elements. A statistically significant relationship was found—those mothers who declared the use of BLW more often use these elements in the process of expanding the child's diet than those who did not make such a declaration.

## Discussion

Balanced nutrition, dietary supplementation, and the nutritional status of the pregnant woman are of great importance in terms of the nutritional status of the child at different stages of psychomotor and neurodevelopmental development. Learning the principles of healthy eating starts at an early age. The development of individual food preferences that consist of learning about and getting used to new tastes contributes to the acquisition of healthy eating habits that will bear fruit in the future. An indispensable component for this is the nutritional knowledge of the parents, who introduce meals to their child in the postnatal period (26, 27).

In the author's study, the degree of women's knowledge was characterized by a high level. The analysis of our results allows us to state that the elements from the scope of knowledge concerning BLW: knowledge of the method, terminology, and the validity of its use constituted more than a good level, at the same time taking into account the extension of this scope of issues using the Internet. Comparing the study of Brown and Lee it shows an analogy with the author's analysis because also the degree of the range of skills? was also at a high level, but the subjects were informed by health professionals (28).

The process of introducing complementary products to breastfeeding as well as for babies fed with modified milk is important for the baby's microbiome. This is due to the type of food products that contain natural prebiotics such as fructo-oligosaccharides and galacto-oligosaccharides. By expanding the baby's diet and introducing products such as yogurt, kefir, buttermilk and products fortified with probiotics we influence the microbiome. By taking advantage of the regionality of product groups and relying on products such as sauerkraut, pickled cucumbers and other pickles, we promote the formation of an immunomodulatory barrier. Popular Polish regional products such as sauerkraut, pickled cucumbers and fermented milk products contain probiotic bacteria. Additionally, products specially enriched with probiotic bacteria can be purchased on the Polish market. It has been shown that probiotic bacteria, through colonization in the intestines of the host, among other things, reduce the risk of growth of potential pathogenic bacteria and affect the anatomical-physical and microbiological barriers of the gastrointestinal tract, as well as affect the immunity of the macroorganism, including the local immunity of the gastrointestinal tract (29–32). It has been found that different strains of bacteria, even though they belong to the same species, can have different effects on the organism, so when selecting bacteria used in probiotics, it is important to choose those that show good clinical effects (31, 33).

Probiotic bacteria show a wide spectrum of action, including stimulating the mucosa associated lymphoid tissue (MALT) immune system—also called the common mucosal immune system (CMIS) (34, 35). The MALT or CMIS system is formed, among others, by elements of immunity of the gastrointestinal tract (GALT—gut associated lymphoid tissue), respiratory system (BALT—bronchus associated lymphoid tissue), genitourinary associated lymphoid tissue (GUALT), and skin SALT—skin associated lymphoid tissue. The immune response of the MALT system, is characterized by activation of natural and acquired immunity mechanisms, through production of a whole range of cytokines, chemokines and growth factors, as well as immunoglobulins class: G, M, A, including secretory immunoglobulins S-IgA and S-IgM (34–37). As the last mentioned antibodies, min. by coating and agglutinating microorganisms, they prevent their adhesion to the gastrointestinal epithelium, although they also show regulatory effects, including through S-IgM antibodies, on elements of the immune system. Thus, the MALT system shows not only a combative or destructive effect against bacteria, viruses, fungi and parasites, but also causes neutralization of bacterial and fungal toxins Probiotics show a significant effect on the elements of the immune system in the gastrointestinal tract (GALT), which results in increased local, but also general immunity, which in turn leads to a reduction in the impact of infections, including bacterial, viral and other on the macroorganism. This effect is combined with and depends on the appropriate strain of bacteria used in the probiotic and this is, it seems, crucial for a good effect in the use of probiotics, although its effect is also influenced by its dose and time of administration, but also by the immune status of the macroorganism (30, 31, 36–41).

WHO and ESPGHAN recommend exclusive breastfeeding at least until the child is 6 months old, whereas after 6 months of age and between 17 and 26 weeks of age, according to PTGHiZD, the use of supplementary products is allowed according to individual decision and needs of the mother and child (26, 42). The knowledge of the principles of dietary expansion of infants translates into the level of frequency of its application. Our study shows the reflection of knowledge in the frequency of BLW implementation by mothers. The universality of this form of dietary expansion is confirmed by the results of a study by other authors, which also allowed to demonstrate a relationship between a higher level of education and an increased frequency of using the method of introducing solid products (28).

Nutritional patterns reflected in the parents' diet are reproduced and implemented in children's nutrition. The mechanism of metabolic programming has been shown to influence the pathomechanism of the development of metabolic and diet-related diseases, the regulation of leptin and ghrelin synthesis in the hypothalamus, and the nutritional status of the child (43, 44). Based on scientific reports, the timing of administration of specific products is important to the occurrence of later eating habits. In the case of sweet products, they should be introduced as late as possible and at least meet 5% of the energy value of the whole-day ration, due to strong taste habits. The researchers Townsend and Pitchford proved the positive influence on health-promoting habits in the case of using the BLW method in comparison with children not eating this way, additionally demonstrating in them an increased inclination to sweet products (14, 45). Additionally, the authors Brown and Lee emphasize the positive and safe quality of food prepared using the BLW method (28).

Our results show the predominance of the mixed form of complementary feeding and the lower frequency of the full BLW method. Among mothers who did not use this method, an increased tendency not to give products from the family table was observed, as well as a more frequent lack of parents' consent to decide independently about the quantity and quality of eaten products.

The analysis of the author's study shows that the dietary model was expanded to include vegetables, fruits in the first place, using favorable thermal treatment including methods such as boiling, steaming, baking, stewing which are the correct techniques for preparing meals according to the nutritional recommendations for children (26).

The variety of controversies arising around the BLW method is conditioned by the disadvantages and advantages attributed to this way of nutrition. The disadvantages include chaos during meal consumption, the possibility of choking, increase in saturated fat supply, nutritional deficiencies, insufficient coverage of nutritional fiber requirements, wasting of food (26, 28). The increased incidence of choking, which is the main concern among parents and health care professionals, is not confirmed according to scientific reports, as it occurs similarly in spoon-fed children and is regulated by the defense mechanism of the developed unconditioned reflex To avoid choking, check the food before serving to make sure it is soft enough. Avoid food that forms crumbs in the mouth or that can stick to the palate. The child should eat at his own pace and under his own control—no one should force the child to eat more quickly. Small foodstuffs, such as chickpeas, grapes and blueberries, should always be mashed first and cut up properly. It is also forbidden to give baby nuts whole, but blended nuts in the form of nut butter, cashew butter or walnut butter can be given. The child should have a stable sitting position while eating—it should not lean back or to the sides. And an adult should always be with the child (45–47).

Among the elements inducing the implementation of BLW, according to the studies of other researchers and in the author's study, the following are important: expansion of family ties through the use of the family table method, the pleasure of meal consumption, the formation of healthy eating habits (increased supply of vegetables, fruit, whole grain products), the taste and organoleptic diversity, the formation of independence in food consumption and the formation of self-control in the relationship with food and, above all, the feeling of satiety (28, 48, 49). It is also worth noting that in our study it was observed that there is a percentage of mothers who, despite the lack of declaration of using the BLW method with their child, implement in feeding their children elements which are a departure from the traditional model of nutrition. Such a relationship is also demonstrated in the study by Brown (50).

It is necessary to implement continuous nutritional education on the nutrition of infants, children, as well as women planning pregnancy, with ongoing pregnancy and during lactation, including elements of psychodietetics based, for example, on motivational dialogue. The principles of anti-inflammatory dietoprophylaxis used among people trying to have a baby are common. The typical anti-inflammatory diet emphasizes fruits, vegetables, lean protein, nuts, seeds, and omega-3 fatty acids. Many products are eliminated from the diet, incl. Foods high in omega-6 fatty acids include: high-fat dairy products (such as milk, cheese, butter, and ice cream), margarine, meats, peanuts. It is important that parents follow healthy eating principles when expanding their child's diet rather than restrictively eliminating foods. Such restrictions may predispose to food aversion, food neophobia and even to avoidant/restrictive food intake disorder (AFRID) (51, 52).

## Conclusions

The level of women's knowledge in the scope of dietary expansion of infants by the BLW method is high, taking into account the necessity of continuous nutritional education. The use of the mixed method of dietary expansion is dominant among the mothers participating in the study. The frequency of implementation of the BLW method depended on the level of education; women with higher education use it more often. Among the positive aspects of using the BLW method, the women surveyed indicate the child's independence, while among the disadvantages, mess, and chaos. There is a group of mothers who, despite not being identified with the BLW method, use its elements in the child's everyday feeding, such as the child deciding on its own how much and what to eat.

## Limitations of the Study

The results of the study should be interpreted taking into account its limitations. Limitations include the low heterogeneity of the study group in terms of age (predominance of women aged 30–35) and education level (predominantly higher education) despite the random selection of nurseries and clinics where data collection took place. Our study was a retrospective study, which may influence the occurrence of false memory effect, especially in the group of mothers of children aged 2–3 years.

The advantage of our study is the size of the study group–699 qualified mothers with their children and the random selection of nurseries and pediatric outpatient clinics where this study was conducted. It is also worth mentioning here that very few studies on this topic have been conducted so far.

## Data Availability Statement

The original contributions presented in the study are included in the article/supplementary material, further inquiries can be directed to the corresponding author.

## Ethics Statement

The studies involving human participants were reviewed and approved by Bioethics Committee of the Medical University of Silesia in Katowice (No. KNW/0022/KB1/151/I/11). Written informed consent to participate in this study was provided by the participants' legal guardian/next of kin.

## Author Contributions

AB-D and PT: conceptualization. PT, AB-D, and MGro: investigation. AB-D, MGro, and MGra: writing-original draft preparation: AB-D, MGro, MGra, ES, and OK: writing-review and editing. AB-D: visualization. ES and OK: supervision. All authors have read and agreed to the published version of the manuscript.

## Conflict of Interest

The authors declare that the research was conducted in the absence of any commercial or financial relationships that could be construed as a potential conflict of interest.

## Publisher's Note

All claims expressed in this article are solely those of the authors and do not necessarily represent those of their affiliated organizations, or those of the publisher, the editors and the reviewers. Any product that may be evaluated in this article, or claim that may be made by its manufacturer, is not guaranteed or endorsed by the publisher.
